# HOXA7 promotes the metastasis of KRAS mutant colorectal cancer by regulating myeloid-derived suppressor cells

**DOI:** 10.1186/s12935-022-02519-9

**Published:** 2022-02-19

**Authors:** Yunzhi Dang, Jiao Yu, Shuhong Zhao, Ximing Cao, Qing Wang

**Affiliations:** Department of Radiation Oncology, Shaanxi Provincial People’s Hospital, Xi’an, 710086 China

**Keywords:** Colorectal cancer, Metastasis, HOXA7, Myeloid-derived suppressor cells

## Abstract

**Background:**

KRAS mutation accounts for 30–50% of human colorectal cancer (CRC) cases. Due to the scarcity of effective treatment options, KRAS mutant CRC is difficult to treat in the clinic. Metastasis is still the major cause of the high mortality associated with KRAS mutant CRC, but the exact mechanism remains unclear. Here, we report a unique function of Homeobox 7 (HOXA7) in driving KRAS mutant CRC metastasis and explore therapeutic strategies for subpopulations of patients with this disease.

**Methods:**

The expression of HOXA7 in a human CRC cohort was measured by immunohistochemistry. The function of HOXA7 in KRAS mutant CRC metastasis was analyzed with the cecum orthotopic model.

**Results:**

Elevated HOXA7 expression was positively correlated with lymph node metastasis, distant metastasis, poor tumor differentiation, high TNM stage, and poor prognosis in CRC patients. Furthermore, HOXA7 was an independent prognostic marker in KRAS mutant CRC patients (P < 0.001) but not in KRAS wild-type CRC patients (P = 0.575). Overexpression of HOXA7 improved the ability of KRAS mutant CT26 cells to metastasize and simultaneously promoted the infiltration of myeloid-derived suppressor cells (MDSCs). When MDSC infiltration was blocked by a CXCR2 inhibitor, the metastasis rate of CT26 cells was markedly suppressed. The combination of the CXCR2 inhibitor SB265610 and programmed death-ligand 1 antibody (anti-PD-L1) could largely inhibit the metastasis of KRAS mutant CRC.

**Conclusions:**

HOXA7 overexpression upregulated CXCL1 expression, which promoted MDSC infiltration. Interruption of this loop might provide a promising treatment strategy for HOXA7-mediated KRAS mutant CRC metastasis.

**Supplementary Information:**

The online version contains supplementary material available at 10.1186/s12935-022-02519-9.

## Introduction

Colorectal cancer (CRC) is the third most frequently diagnosed cancer and the second leading cause of cancer-related death in the world [1]. The 5-year survival rate for patients with metastatic CRC is only 12–14%. KRAS mutation is associated with disease aggressiveness and metastasis and appears in 30–50% of human CRC patients [2, 3]. For metastatic CRC patients with wild-type KRAS, anti-EGFR antibodies, such as cetuximab or panitumumab, in conjunction with chemotherapy, are effective treatment options. However, the clinical efficacy of these treatments in KRAS mutant CRC patients is often poor because of the intrinsic drug resistance generated by KRAS mutation [4]. Bevacizumab, a recombinant humanized VEGF-A-targeting monoclonal antibody, is recommended for stage IV CRC treatment combined with other cytotoxic agents, but the response rate is still low [5]. In addition, KRAS mutation is associated with suppressed Th1/cytotoxic immunity in CRC patients [6], emphasizing that treating this subset of patients remains a challenge. Sotorasib, a novel KRAS^G12C^ specific inhibitor, covalently binds to a pocket of the switch II region of KRAS^G12C^ which is present only in the inactive GDP-bound conformation. This binding maintains KRAS^G12C^ in its inactive state and inhibits KRAS oncogenic signaling [7]. Sotorasib has been approved by the FDA to treat non-small cell lung cancer (NSCLC) with the KRAS^G12C^ mutation. However, the effective rate in patients with CRC with the KRAS^G12C^ mutation is poor [8], and the underlying mechanism is unclear. Recently, researchers discovered that combining sotorasib with anti-PD-1 antibodies results in a long-lasting antitumor response [9], suggesting that combined therapy might be a potential therapeutic option for subpopulations of patients with KRAS mutant CRC.

The Homeobox (HOX) transcription factor family plays a critical role in embryonic development and regulates various cellular processes [10]. In recent years, increasing evidence has shown that dysregulation of HOX genes enhances cancer initiation and progression via different mechanisms [11]. The HOX family in mammals is composed of 39 proteins that are divided into four groups (A, B, C, and D). HOXA subfamily genes are critical regulators of cancer metastasis. Homeobox 7 (HOXA7), a member of the HOXA subfamily, has been shown to function as an oncogene and contributes to malignant behaviors in various human cancers, including hepatocellular carcinoma [12], laryngeal squamous cell cancer [13], and ovarian cancer [14]. However, the function of HOXA7 in CRC metastasis is poorly understood.

The infiltration of relatively immature and pathologically activated myeloid-derived suppressor cells (MDSCs) with significant immunosuppressive function is frequently observed in malignancies. The accumulation of MDSCs, which cause immunosuppression, can promote tumor progression [15]. In addition, MDSCs can directly promote tumor cell survival, angiogenesis, invasion, and metastasis [16]. Microsatellite instability-low (MSI-L) CRC is more extensively infiltrated by Tregs and MDSCs. Additionally, T cell infiltration is significantly reduced, which is the leading cause of the poor effectiveness of immune checkpoint blockade [17, 18]. A previous study showed considerable differences in immune cell infiltration and the expression of immune-related markers between KRAS mutant CRC and KRAS wild-type CRC. KRAS mutant CRC, for example, has a decreased T helper 1 (Th1)-centric coordinated immune response cluster and reduced cytotoxic cell infiltration [6]. MDSC infiltration is significantly increased, and MDSC depletion may markedly improve anti-programmed death ligand 1 (anti-PD-L1) efficacy [19, 20]. However, the haptic oncogenic signal in KRAS mutant CRC that stimulates MDSC recruitment and activation is still poorly understood.

Here, we demonstrated that HOXA7 expression was upregulated and associated with poor prognosis in patients with KRAS mutant CRC. Overexpression of HOXA7 promoted KRAS mutant CRC metastasis by upregulating CXCL1 expression. The CXCR2 inhibitor SB265610 combined with anti-PD-L1 treatment significantly reduced HOXA7-mediated KRAS mutant CRC metastasis.

## Materials and methods

### Cells and culture

All cell lines were purchased from The American Type Culture Collection (ATCC), Manassas, VA, USA. CT26, a murine colon cancer cell line, was cultured in Dulbecco’s Modified Eagle Medium (DMEM, Gibco, Rockville, MD, USA) medium. The medium was supplemented with 10% FBS (Gibco), 100 μg/ml penicillin, and 100 μg/ml streptomycin. The cell lines SW620, HCT116, Lovo, HT29, CaCo-2, HCA-7 were cultured in RPMI-1640 (Gibco) medium with 10% FBS (Gibco), 100 μg/ml penicillin, and 100 μg/ml streptomycin.

### Immunohistochemistry

This study was approved by the ethics the Committee of Shaanxi Provincial People’s Hospital, and informed consent was written and based on the ethical guidelines of the 1975 Declaration of Helsinki. Written informed consent was obtained from all patients. Furthermore, human subjects’ private rights were routinely respected. CRC specimens and matched adjacent tissues were used to construct a tissue microarray (Shanghai Biochip Co, Ltd. Shanghai, China). The tissue microarray was stained for HOXA7 (Abcam, ab211521), CD11b (Abcam, ab6640), CD8 (Abcam, ab4055) expression.

Paraffin-embedded sections were continuously sliced when cutting the tissue by microtome. Immunohistochemistry was carried out on 4-μm thick paraffin-embedded sections, and was performed according to standard procedures as outlined in our previous study [21]. Briefly, after baking on a panel at 60 °C for an hour, the tissue sections were deparaffinized with xylene and rehydrated through gradient ethanol immersion. Endogenous peroxidase activity was quenched by 3% (vol/vol) hydrogen peroxide in methanol for 12 min, followed by three 3-min washes with phosphate-buffered saline (PBS). Then the slides were immersed in 0.01 mol/l citrate buffer solution (pH = 6.0) and placed in a microwave oven for 30 min. After washing in PBS, the sections were incubated in a moist chamber at 4 °C overnight with the primary antibody diluted in PBS containing 1% (wt/vol) bovine serum albumin. Negative controls were performed by replacing the primary antibody with preimmunized mouse serum. After three 5 min washes with PBS, the sections were treated with a peroxidase-conjugated second antibody (Santa Cruz) for 30 min at room temperature, followed by an additional three 5 min washes with PBS. The reaction product was visualized with diaminobenzidine for 2 min. Images were obtained under a light microscope (Olympus, Japan) equipped with a DP70 digital camera.

Analyses were performed by two independent observers who were blinded to the clinical outcome. The immunostaining intensity was scored on a scale of 0 to 3: 0 (negative), 1 (weak), 2 (medium), or 3 (strong). The percentage of positive cells was evaluated on a scale of 0 to 4: 0 (negative), 1 (1–25%), 2 (26–50%), 3 (51–75%), or 4 (76–100%). The final immuno-activity scores were calculated by multiplying the above two scores, resulting in an overall score of 0–12. Each case was ultimately considered “negative” if the final score ranges from 0 to 3 and “positive” if the final score ranges from 4 to 12.

### In vivo metastatic model and bioluminescent imaging

Our implantation tests were under the approval of the ethics of the Committee of Shaanxi Provincial People’s Hospital. All the mice were male. All efforts were made to minimize the animals’ suffering during the experiments. BALB/C mice (5 weeks old) were housed under standard conditions and cared for according to the institutional guidelines for animal care. A metastatic colorectal cancer model in mice was established according to the existing protocol. Luciferase labeled mouse CRC cells (4.0 × 10^6^) were injected into the cecal wall in mice under anesthesia (n = 10 for each group). Briefly, the caecum was gently exteriorized and was placed on a scalpel holder, flattened, and stabilized with forceps. This maneuver is crucial to prevent leakage of tumor cells into the caecal lumen or peritoneal cavity. A volume of 100 μl (4.0 × 10^6^) cells was injected into the caecal wall. Then, the caecum was returned to the peritoneal cavity, peritoneum and skin were closed by running sutures and wound clips.

To investigate whether SB265610 can improve CRC responsiveness to anti-PD-L1 blocking in KRAS mutant CRC, SB265610 (2 mg/kg body weight, i.p. every day), or anti-PD-L1 antibody (200 μg, i.p., every three days), or Vehicle (i.p., every day) was injected. For combined treatment, mice were treated with SB265610 (2 mg/kg body weight, i.p. every day) and anti-PD-L1 antibody (200 μg, i.p., every three days).

Luciferase lentivirus was purchased from Shanghai Genechem Co., Ltd. Concentrated luciferase lentivirus was transfected into the CRC cells with a multiplicity of infection (MOI = 50) in the presence of polybrene (6 μg/ml). Seventy-two hours after infection, CRC cells were selected for 2 weeks using 2.5 μg/ml puromycin (OriGene).

The in vivo tumor formation and metastases were imaged by bioluminescence. d-luciferin (Xenogen, Hopkinton, MA) at 100 mg/kg was injected intraperitoneally into the mice. To anesthetize the mice, the mice were inhaled 2.5% isoflurane in the special volatilization chamber for 5–6 min. Then, bioluminescence was detected using an IVIS 100 Imaging System (Xenogen). After acquiring photographic images of each mouse, luminescent photos were captured using various (1–60 s) exposure times. The resulting grayscale photographic and pseudocolored luminescent images were automatically superimposed using the IVIS Living Image (Xenogen) software. This superimposition was performed to facilitate the matching of the observed luciferase signal with its location on the mouse. The survival of the mice was recorded daily.

At the 9 weeks, the mice were sacrificed by injecting excessive pentobarbital sodium for anesthesia (100 mg/kg, Merck, Germany), and the livers and lungs were collected and underwent histological examination.

### Preparation of single cell suspensions

Mice were perfused with PBS and anesthetized, and tumors were dissected using a clean razor. Then, the tumor tissues were digested with DNase I (20 mg/ml, Sigma-Aldrich) and collagenase IV (1.5 mg/ml, Sigma-Aldrich) and placed on a thermomixer (Thermo Scientific), 37 °C, 40 rpm, for 1 h to digest the tissue adequately. At the end of 1 h, we filtered the dissociated cells through 70 μm pore filters rinsed with fresh media. Cell staining buffer was added to the separation tube to 15 ml, then centrifuged at 350*g* for 5 min, and the supernatant was discarded. The red cell lysis (Biolegend, #420301) was added to pelleted disintegrated tissue and incubated for 5 min to lysis the red blood cell. Cell staining buffer (10 ml) was added to the tube to stop lysis. The supernatant was then discarded after centrifugation at 350*g* for 5 min, and repeat this step again.

### Flow cytometric analysis

Cells were incubated with anti-mouse CD16/CD32 purified antibody (#101302, clone 93, Biolegend) for 10 min to block nonspecific antibodies. Then, the cells were stained with fluorophore-conjugated antibodies. Matched isotype antibodies were used as control. All antibodies were purchased from Biolegend. Antibodies against CD45 (PE, #103105, 0.25 µg/10^6^ cells), CD11b (FITC, #101205, 0.25 µg/10^6^ cells), CD45 (PE/Cy7, #103113, 0.30 µg/10^6^ cells), Ly-6G/Ly-6C (Gr-1) (PE, #108407, 0.25 µg/10^6^ cells), CD3 (FITC, #100203, 1.0 µg/10^6^ cells), CD8 (PE, #100707, 0.20 µg/10^6^ cells) were used. Data were analyzed by Flowjo_V10 software (TreeStar, Ashland, OR).

### Statistical analysis

Statistics were calculated with SPSS software (version 20.0). P values were statistically analyzed by the χ^2^ test for categorical variables and by Student’s test for quantitative data. The recurrence and survival data were analyzed by the Kaplan–Meier method. Cox proportional hazards model was used for univariate and multivariate analyses. Differences were considered statistically significant when P < 0.05.

## Results

### Elevated HOXA7 positively correlates with poor prognosis in CRC patients harboring KRAS mutation

**Fig. 1:**
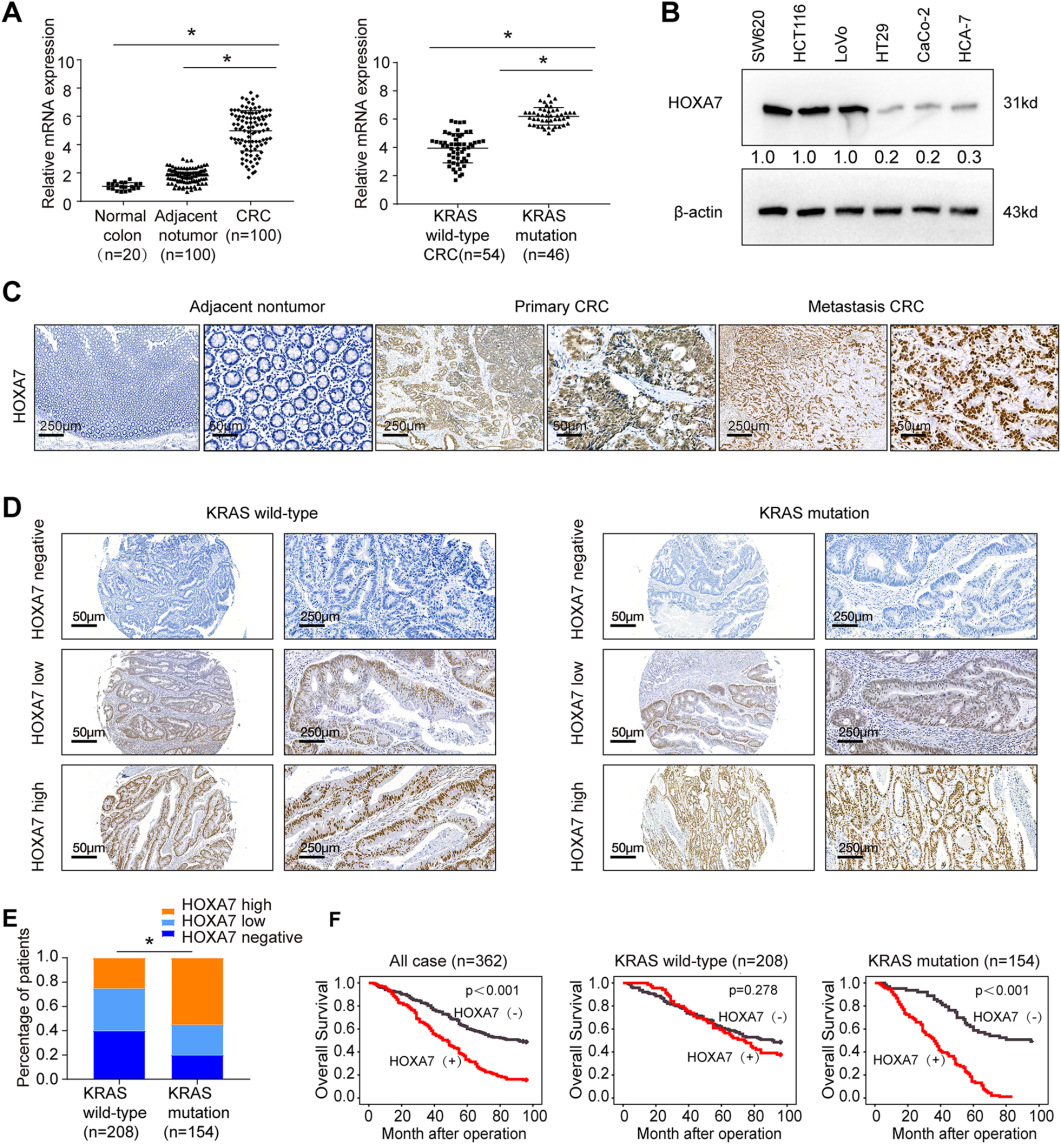
Elevated HOXA7 expression is positively correlated with poor prognosis in KRAS mutant CRC. **A** Relative HOXA7 mRNA expression in 20 normal colon tissues and 100 paired CRC and adjacent nontumorous tissues (left). Relative HOXA7 mRNA expression in CRC tissues expressing wild-type KRAS or mutant KRAS (right). **B** Western blotting analysis of HOXA7 expression in KRAS mutant CRC cell lines (SW620, HCT116, and LOVO) and KRAS wild-type CRC cell lines (HT29, Caco-2, HCA-7). **C** Representative IHC images of HOXA7 expression in paired adjacent nontumor tissues, primary CRC tissues, and metastatic CRC tissues. **D** Representative IHC images of HOXA7 expression in KRAS wild-type CRC (left) and KRAS mutant CRC (right). **E** The rate of high HOXA7 expression, low HOXA7 expression and negative HOXA7 expression in KRAS wild-type CRC and KRAS mutant CRC. **F** Kaplan–Meier analysis of the correlation of HOXA7 expression with overall survival in the human CRC cohort (left), KRAS wild-type CRC (middle), and KRAS mutant CRC (right)

To characterize the function of HOXA7 in CRC, we examined its mRNA expression in 20 normal colorectal epithelial specimens and 100 paired CRC and adjacent nontumor specimens. We found that CRC tissues exhibited higher HOXA7 mRNA levels than paired nontumor tissues and normal colorectal epithelial tissues (Fig. 1A, left). To determine the relationship between HOXA7 expression and KRAS mutation status, we performed a KRAS mutation test and discovered that 46% (46 of 100) of CRC patients harbored KRAS mutations. Notably, the HOXA7 mRNA level in patients with mutant KRAS was significantly higher than that in patients with wild-type KRAS (Fig. 1A, right). We next investigated the expression level of HOXA7 in established human CRC cells. HOXA7 expression was higher in KRAS mutant CRC cell lines (SW620, HCT116, and Lovo) than in KRAS wild-type CRC cell lines (HT29, CaCo-2, and HCA-7) (Fig. 1B).

To investigate the role of HOXA7 in human CRC metastasis, immunohistochemistry was used to measure its expression in 20 paired primary and metastatic CRC tissues. A higher level of HOXA7 expression was observed in metastatic CRC tissues than in primary CRC tissues and adjacent nontumor tissues (Fig. 1C). We then aimed to profile HOXA7 expression in 362 samples with a CRC tissue microarray. IHC staining was significantly stronger in KRAS mutant CRC samples than in KRAS wild-type CRC samples (Fig. 1D, E). The overexpression of HOXA7 showed a positive correlation with tumor differentiation, lymph node metastasis, distant metastasis, high tumor nodule metastasis (TNM) stage, and KRAS mutation (Table 1). Elevated HOXA7 expression was an independent predictor of poorer overall survival according to a multivariate analysis (Table 2). When the cohort was divided into KRAS mutant and KRAS wild-type patients, HOXA7 was shown to be an independent prognostic marker in KRAS mutant CRC (P < 0.001, Additional file 1: Table S1) but not in KRAS wild-type CRC (P = 0.575, Additional file 1: Table S2). Patients with positive HOXA7 expression had a shorter overall survival time than those with negative HOXA7 expression, according to the Kaplan–Meier curve. Furthermore, stratification analysis revealed that HOXA7 expression was correlated with poor prognosis in KRAS mutant CRC patients but not in KRAS wild-type CRC patients (Fig. 1F). These studies show that HOXA7 overexpression is a predictive factor in KRAS mutant CRC.

**Table 1:** Correlation between HOXA7 expression and clinicopathological characteristics in a cohort of human CRC

Clinicopathological variables	Tumor HOXA7 expression		*P* value
	Negative (n = 203)	Positive (n = 159)	
Age			
≤ 50	26	28	0.203
> 50	177	131	
Gender			
Female	88	73	0.626
Male	115	86	
Tumor size (cm)			
≤ 5	90	64	0.435
> 5	113	95	
Tumor differentiation			
Well or moderate	158	91	< 0.001
Poor	45	68	
Tumor invasion			
T1–T3	161	116	0.157
T4	69	103	
Lymph node metastasis			
Absent	177	117	0.001
Present	22	17	
Distant metastasis			
Absent	134	56	0.001
Present	69	103	
TNM stage			
I–II	131	59	< 0.001
III–IV	72	100	
KRAS status			
Wild	140	68	< 0.001
Mutation	63	91

**Table 2:** Univariate and multivariate analysis of factors associated with survival and recurrence in a cohort of human CRC (overall population)

Clinical variables	Univariate COX regression analysis		Multivariate COX regression analysis	
	HR (95% CI)	P value	HR (95% CI)	P value
Age (≤ 50 vs > 50)	1.065 (0.750–1.512)	0.726		
Gender (female vs male)	1.142 (0.885–686)	0.349		
Tumor size (≤ 5 cm vs > 5 cm)	1.278 (0.985–1.658)	0.065		
Tumor differentiation (well/moderate vs poor)	0.185 (0.141–0.242)	< 0.001	0.733 (0.522–1.030)	0.073
Tumor invasion (I–II vs III)	0.234 (0.262–0.455)	< 0.001	0.570 (0.420–0.774)	< 0.001
Lymph node metastasis (absent vs present)	0.122 (0.070–0.132)	< 0.001	0.560 (0.313–1.002)	0.051
Distant metastasis (absent vs present)	0.155 (0.114–0.211)	< 0.001	0.508 (0.351–0.737)	< 0.001
TNM stage (I/II vs III/IV)	0.096 (0.114–0.211)	< 0.001	0.232 (0.131–0.412)	< 0.001
HOXA7 expression (negative vs positive)	0.418 (0.322–0.541)	< 0.001	0.724 (0.552–0.948)	0.019

### Elevated expression of HOXA7 promotes KRAS mutant CRC metastasis in immunocompetent mice

To understand the function of HOXA7 in KRAS mutant CRC cells, we evaluated the effect of HOXA7 on cell invasion and migration in loss-of-function experiments both in vitro and in vivo*.* Transwell assays showed that knockdown of HOXA7 expression failed to suppress the invasion and migration abilities of SW620 cells (Additional file 1: Fig. S1A). Furthermore, knockdown of HOXA7 expression had little effect on the rate of SW620 cell metastasis in a nude mouse cecal metastasis model (Additional file 1: Fig. S1B–D). These results indicated that HOXA7 is not required for the migration of CRC in immune-deficient nude mice.

**Fig. 2:**
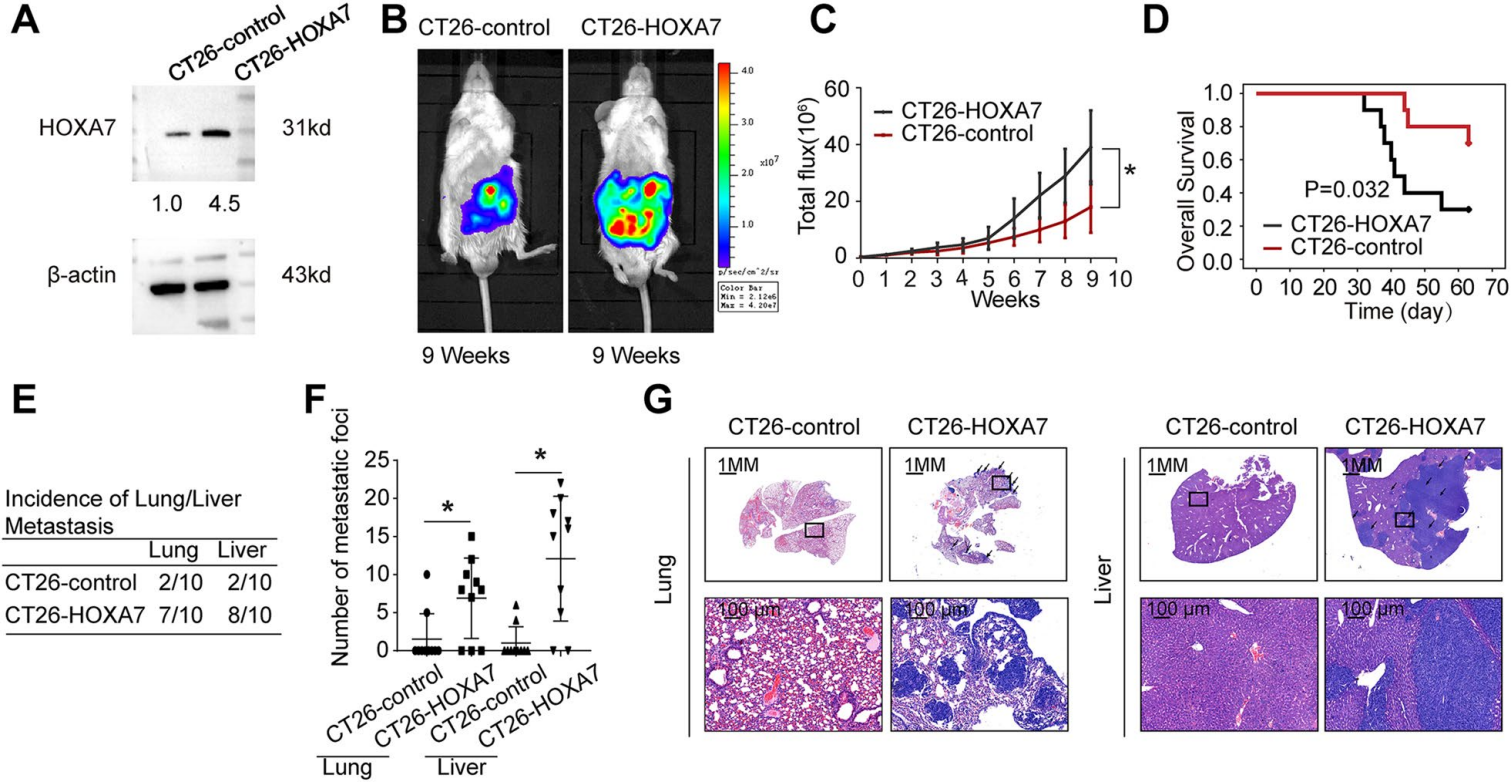
HOXA7 upregulation promotes KRAS mutant CRC metastasis in immunocompetent mice. **A** Western blotting analyses of HOXA7 expression, and the number indicates relative gray values. **B**–**G** In vivo assays showing that HOXA7 overexpression can promote KRAS mutant CRC metastasis. **B** Bioluminescent images showing the presence of metastases in mice 9 weeks after implantation with CT26-HOXA7 cells and CT26-control cells. **C** Bioluminescent signals in each group. **D** Overall survival of the BALB/C mice in each group. **E** The incidence of lung and liver colonization. **F** The number of lung and liver metastatic nodules. **G** HE staining was used to identify metastatic lung and liver nodules at 9 weeks. *P < 0.05

CRC most commonly develops due to persistent colon inflammation and dysregulation of the immune system. Thus, we postulated that HOXA7 might accelerate the course of CRC metastasis by altering the immunological microenvironment. We stably upregulated HOXA7 expression by lentivirus transduction of CT26 cells, which express mutant KRAS. HOXA7 was overexpressed in CT26-luciferase cells as showed by Western blotting studies (Fig. 2A). Overexpression of HOXA7 enhanced tumor growth as indicated by increased intensity of the bioluminescent signal (Fig. 2B, C). Histological analysis confirmed that upregulated HOXA7 expression can increase the rates of liver and lung metastases and the numbers of metastatic nodules (Fig. 2E–G). In addition, HOXA7 reduced the overall survival of BALB/C mice (Fig. 2D). These results suggested that overexpression of HOXA7 can promote KRAS mutant CRC metastasis in immunocompetent mice.

### HOXA7 promotes KRAS mutant CRC metastasis and increases the infiltration of MDSCs

**Fig. 3:**
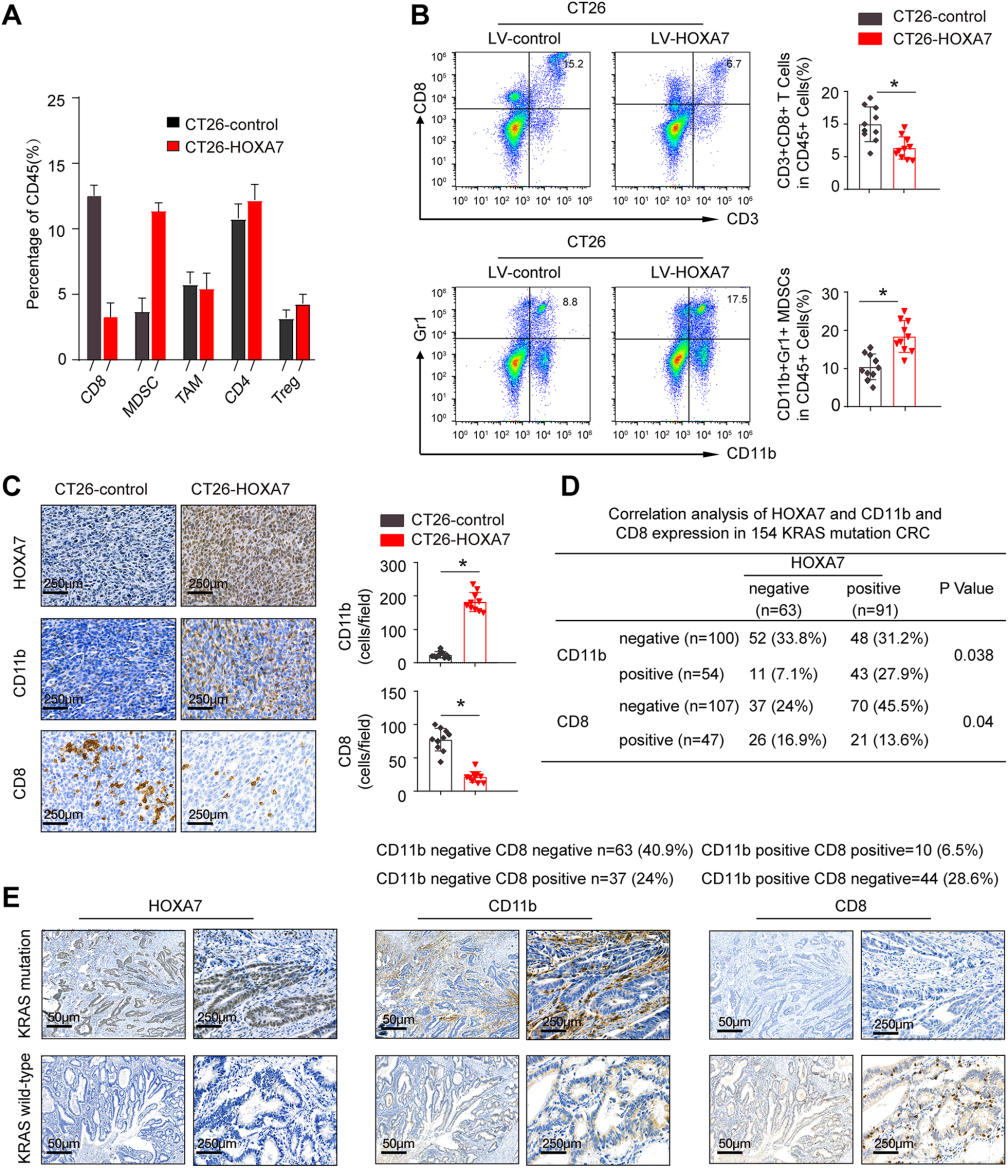
HOXA7 promotes KRAS mutant CRC metastasis and increases the infiltration of MDSCs. **A**, **B** The infiltration of immune cells into CT26-control and CT26-HOXA7 tumors (n = 10 for each group) was analyzed by flow cytometry. **C** The expression of HOXA7 and infiltration of MDSCs and CD8^+^ T cells in the two groups were analyzed by IHC. **D** The correlation between HOXA7 expression and CD11b or CD8 expression in KRAS mutant CRC tissues. The upper P values represent the results of comparing the infiltration of CD11b^+^ cells in the HOXA7-negative group and HOXA7-positive group. The lower P values represent the results of comparing the infiltration of CD8^+^ T cells in the HOXA7-negative group and HOXA7-positive group. **E** IHC staining showing HOXA7, CD11b, and CD8 expression in KRAS mutant CRC and KRAS wild-type CRC tissues

To explore the underlying mechanisms by which HOXA7 signaling enhances KRAS mutant CRC metastasis, we investigated the cellular immune response. The percentage of tumor-infiltrating immune and inflammatory cells was assessed by flow cytometric analysis. In comparison to CT26-control cells, the transplantation of CT26-HOXA7 cells significantly increased MDSC (identified as CD45^+^/CD11b^+^/Gr1^+^) infiltration and decreased the accumulation of CD8^+^ T cells (identified as CD45^+^/CD3^+^/CD8^+^). On the other hand, the numbers of other immune cells, such as CD4^+^ T cells, regulatory T (Treg) cells, and tumor-associated macrophages (TAMs), did not change significantly (Fig. 3A, B). In addition, IHC staining showed that MDSC infiltration was markedly elevated while CD8^+^ T cell infiltration was decreased in CT26-HOXA7 tumors (Fig. 3C). We further evaluated the expression of HOXA7, CD11b, and CD8 in the CRC cohort. Consistent with the above results, IHC staining revealed that HOXA7 expression was positively associated with CD11b expression but negatively related to CD8 expression in CRC patients with KRAS mutations (Fig. 3D, E).

### HOXA7 overexpression induces MDSC chemotaxis through the HOXA7-CXCL1 axis in KRAS mutant CRC

To investigate the mechanism by which HOXA7 recruits MDSCs, we selected KRAS mutant SW620 CRC cell lines with high HOXA7 expression and constructed stable SW620-shHOXA7 cell lines by lentivirus transduction. Using an Affymetrix PrimeView Human Gene Expression Array, we examined changes in the transcriptome of SW620-shHOXA7 and SW620-shcontrol cells. The expression of numerous metastasis-related genes, including CXCL1, OPN, and CXCR2, was reduced when HOXA7 expression was downregulated (Additional file 1: Table S3). CXCL1 recruits MDSCs by interacting with its receptor, CXCR2 [22, 23]. Previous research has shown that CXCL1 produced by tumor cells enhances MDSC recruitment and infiltration to the tumor site, facilitating CRC metastasis [15]. Owing to the pivotal role of CXCL1 in cancer progression, we focused on CXCL1 for further study.

**Fig. 4:**
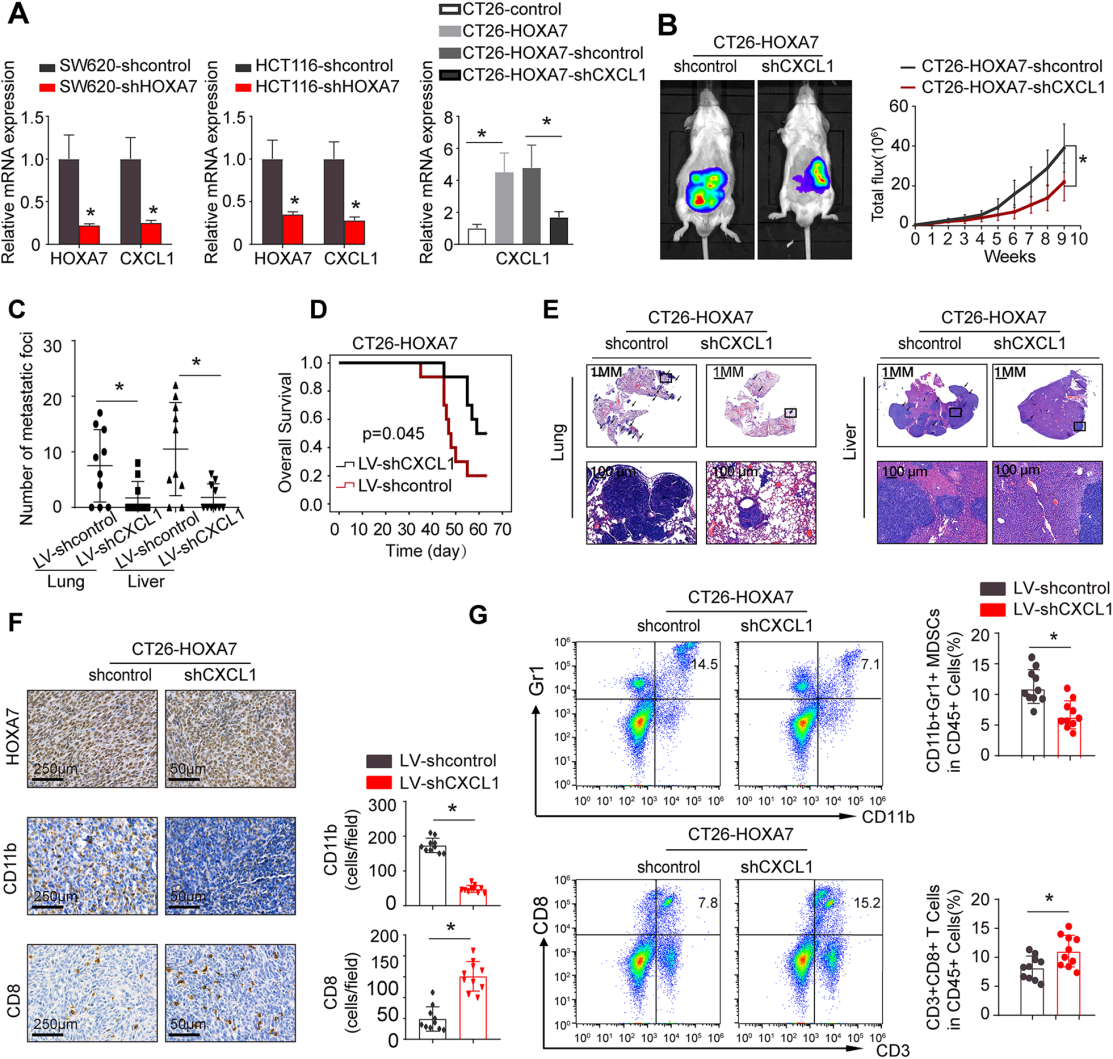
Downregulation of CXCL1 expression can inhibit HOXA7-mediated KRAS mutant CRC metastasis. **A** HOXA7 and CXCL1 expression in the indicated cells as determined by real-time PCR analysis (the Ct value for the indicated gene was shown in Table 3). **B**–**E** CXCL1 knockdown can reduce HOXA7-mediated KRAS mutant CRC metastasis in CT26-HOXA7 cells. **B** Bioluminescence images and bioluminescence signals in the CT26-HOXA7-shcontrol and CT26-HOXA7-shCXCL1 groups at 9 weeks. **C** The number of lung and liver metastatic nodules in each group at 9 weeks. **D** Overall survival of the BALB/C mice in each group. **E** HE staining was used to identify metastatic lung and liver nodules at 9 weeks. **F** The expression of HOXA7, CD11b and CD8 of primary tumor in CT26-HOXA7-shcontrol and CT26-HOXA7-shCXCL1 groups was analyzed by IHC. **G** The infiltration of MDSCs and CD8^+^ T cells in CT26-HOXA7-shcontrol and CT26-HOXA7-shCXCL1 groups was analyzed by flow cytometry. All the data are presented as the mean ± s.d. *P < 0.05

Real-time assays showed that downregulation of HOXA7 expression markedly reduced CXCL1 expression in both SW620 and HCT116 cells (Fig. 4A). To study the function of CXCL1 in CRC metastasis, we downregulated CXCL1 expression in CT26-HOXA7 cells by lentivirus transduction. Endogenous CXCL1 expression was downregulated in CT26-HOXA7-shCXCL1 cells, as shown by RT-PCR (Fig. 4A). Then, we established a cecal metastasis model using CT26-HOXA7-shcontrol cells and CT26-HOXA7-shCXCL1 cells in BALB/C mice. An in vivo study showed that CXCL1 knockdown significantly reduced the lung and liver metastasis rates and prolonged the overall survival of the CT26-HOXA7 group compared to the control group (Fig. 4B–E). Moreover, IHC staining and flow cytometric analysis showed that knockdown of CXCL1 expression in CT26-HOXA7 cells significantly reduced the infiltration rate of MDSCs while increasing the infiltration rate of CD8^+^ T cells compared with the control (Fig. 4F, G). Taken together, these results indicated that HOXA7 promotes KRAS mutant CRC metastasis and MDSC chemotaxis through the HOXA7-CXCL1 axis.

**Table 3:** The Ct value for the indicated gene

Cell type	Gene	Ct value
SW620-shcontrol	β-actin	14
SW620-shcontrol	HOXA7	18
SW620-shHOXA7	β-actin	15
SW620-shHOXA7	HOXA7	21
HCT116-shcontrol	β-actin	15
HCT116-shcontrol	HOXA7	16
HCT116-shHOXA7	β-actin	15
HCT116-shHOXA7	HOXA7	18
SW620-shcontrol	β-actin	14
SW620-shcontrol	CXCL1	20
SW620-shHOXA7	β-actin	15
SW620-shHOXA7	CXCL1	23
HCT116-shcontrol	β-actin	15
HCT116-shcontrol	CXCL1	16
HCT116-shHOXA7	β-actin	16
HCT116-shHOXA7	CXCL1	19

### Depletion of MDSCs can decrease HOXA7-mediated KRAS mutant CRC metastasis

**Fig. 5:**
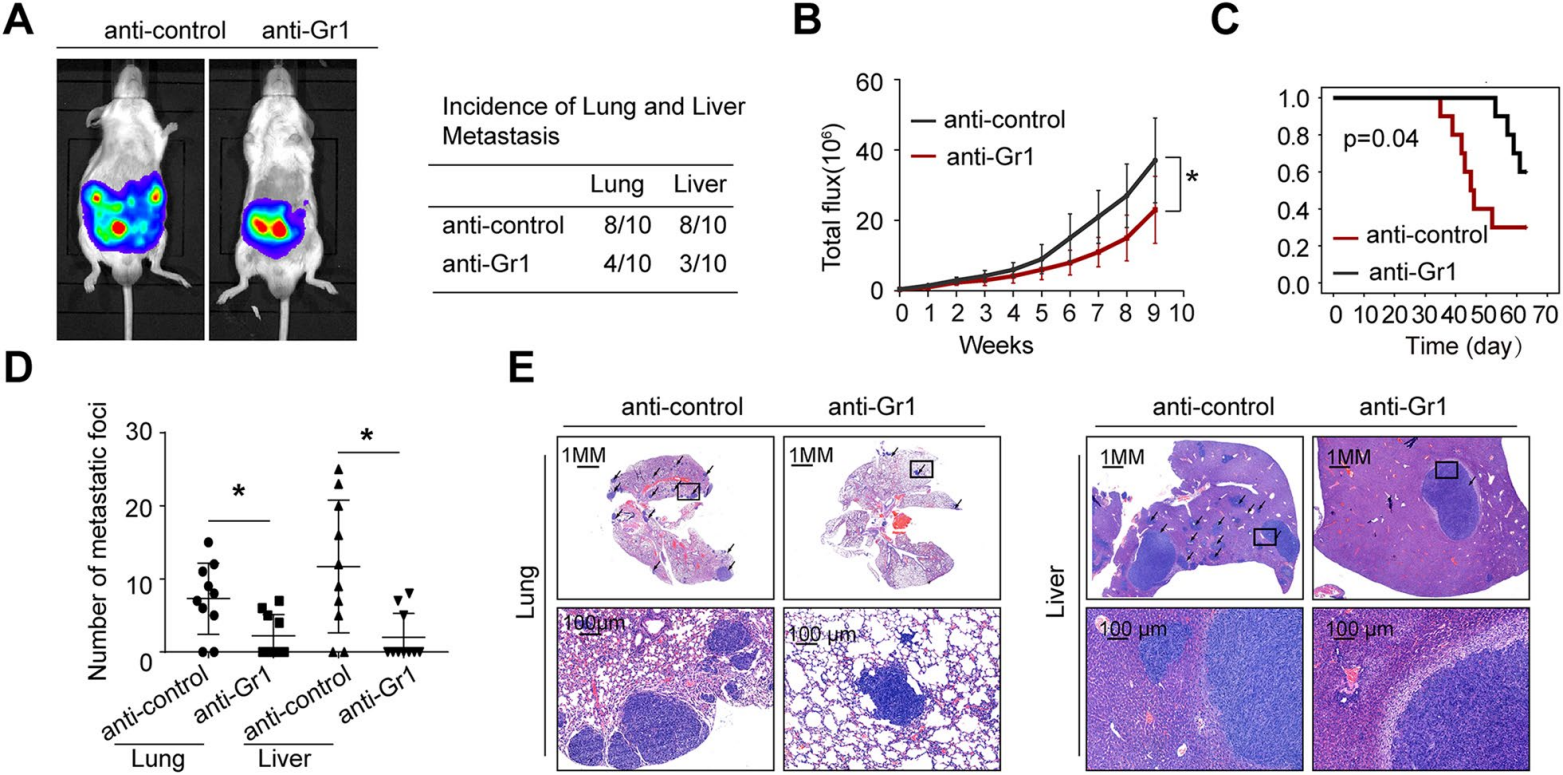
Depletion of MDSCs by anti-Gr1 can decrease HOXA7-induced KRAS mutant CRC metastasis. **A**–**E** In vivo administration of the anti-Gr1 neutralizing monoclonal antibody (clone RB6-8C5, 200 μg/mouse, i.p., every 3 days) or isotype control until the treatment endpoint in CT26-HOXA7 cells. Anti-Gr1 antibody treatment can significantly inhibit HOXA7-mediated KRAS mutant CRC metastasis. **A** Bioluminescence images and the incidence of lung and liver metastasis in the vehicle and anti-Gr1 groups. **B** Bioluminescence signals in each group. **C** Overall survival of the BALB/C mice in each group. **D** The numbers of lung and liver nodules. **E** HE staining was used to identify metastatic lung and liver nodules at 9 weeks. *P < 0.05

The enrichment of MDSCs in CT26-HOXA7 cell orthotopic transplantation tumors prompted us to study the specific role of MDSCs in HOXA7-mediated KRAS mutant CRC metastasis. Then, we established a cecal metastasis model using CT26-HOXA7 cells in BALB/C mice. We depleted MDSCs with a neutralizing monoclonal antibody (clone RB6-8C5), a well-characterized anti-Gr1 antibody [24]. An in vivo metastasis assay showed that HOXA7 upregulation enhanced lung and liver metastasis and decreased the survival time of BALB/C mice. Anti-Gr1 antibody treatment decreased the incidence of lung and liver metastasis while extending the overall survival time of the CT26-HOXA7 group (Fig. 5A–E). Together, these results strongly support the view that MDSCs play a pivotal role in HOXA7-mediated KRAS mutant CRC metastasis.

### Combined application of the CXCR2 inhibitor SB265610 and anti-PD-L1 antibody treatment significantly blocks HOXA7-mediated KRAS mutant CRC metastasis

**Fig. 6:**
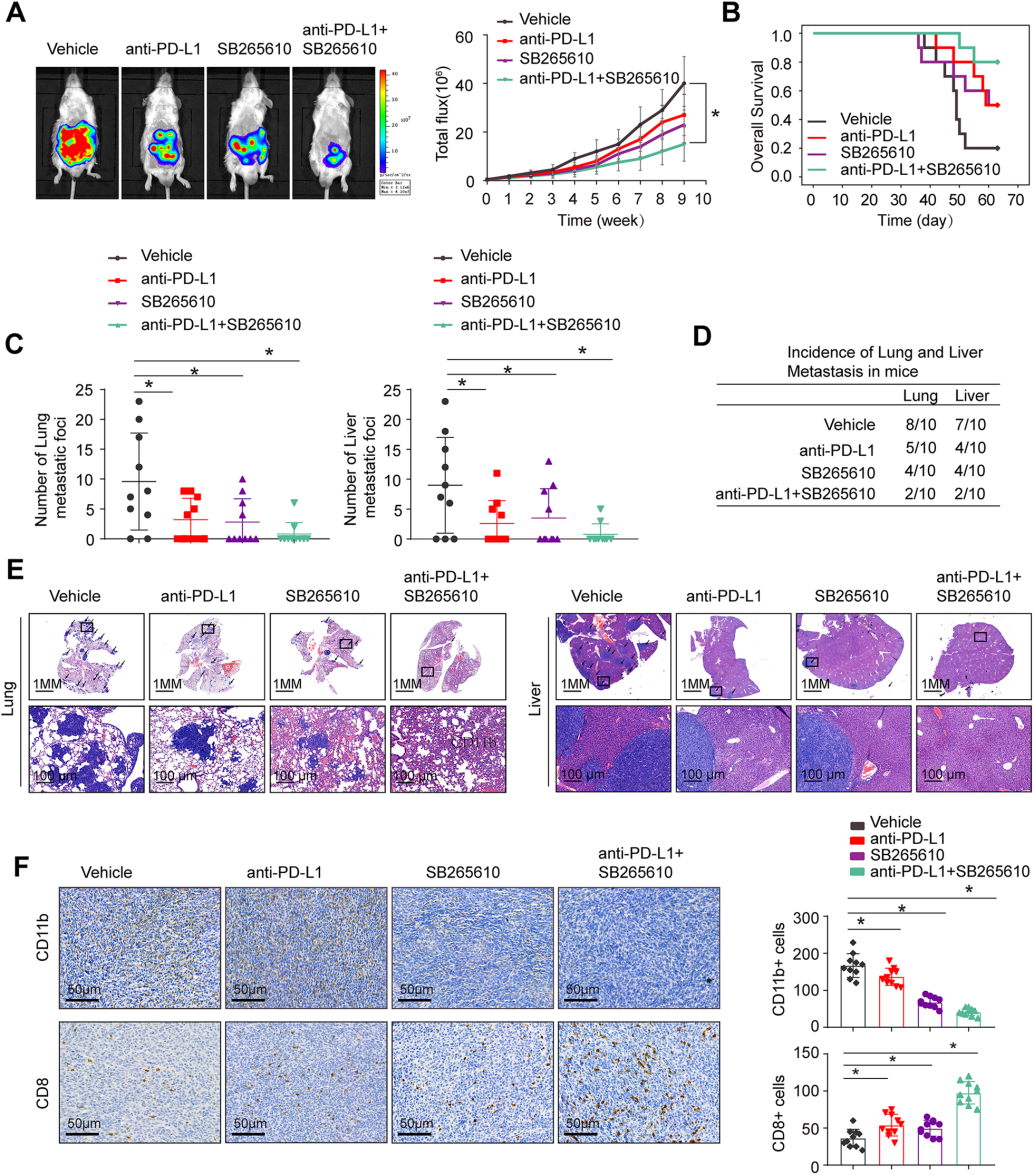
Combined application of the CXCR2 inhibitor SB265610 and anti-PD-L1 antibody treatment significantly blocks HOXA7-promoted KRAS mutant CRC metastasis. **A**–**E** One week after the injection of CT26-HOXA7 cells, the mice in the four groups were treated with vehicle, PD-L1 antibody or/and SB265610 (n = 10 mice/group) until the treatment endpoint. In vivo assays showing that combined treatment with an anti-PD-L1 antibody and the CXCR2 inhibitor SB265610 almost completely blocked KRAS mutant CRC metastasis. **A** Representative bioluminescent images and bioluminescent signals in the indicated groups. **B** Overall survival in the indicated groups. **C** The numbers of lung and liver nodules in the indicated groups. **D** The incidence of lung and liver nodules in the indicated groups. **E** Representative HE staining of lung and liver tissues. **F** IHC staining showing the infiltration of MDSCs or CD8^+^ T cells of primary tumor in the indicated groups. All the data are presented as the mean ± s.d. *P < 0.05

Depletion of MDSCs was shown to have a synergistic impact with anti-PD-L1 in CRC [25, 26]. SB265610, a selective CXCR2 inhibitor, has been shown to decrease the recruitment and infiltration of MDSCs and TAMs in CRC [27]. We wondered whether SB265610 can improve CRC responsiveness to anti-PD-L1 blockade in KRAS mutant CRC. Thus, we established a cecal metastasis model using CT26-HOXA7 cells in BALB/C mice. In vivo metastatic assays revealed that SB265610 or anti-PD-L1 therapy alone could reduce the incidence of lung and liver metastasis and metastatic nodule numbers, as well as extend the overall survival time of CT26-HOXA7 mice. Compared to SB265610 or anti-PD-L1 alone, the combination of SB265610 and anti-PD-L1 significantly reduced lung and liver metastasis rates and metastatic nodule numbers, as well as essentially extended survival time of BALB/C mice (Fig. 6A–E).

To investigate the mechanism underlying the antitumor response triggered by the combination of SB265610 and anti-PD-L1 antibody, we detected the infiltration of MDSCs and CD8^+^ T cells in CT26-HOXA7 orthotopic CRC tumors. In the combined therapy group, IHC staining revealed substantially decreased MDSC infiltration and significantly increased CD8^+^ T cell infiltration (Fig. 6F). These results indicated that double blockade of PD-L1 and SB265610 increased the antitumor response and suppressed HOXA7-mediated KRAS mutant CRC metastasis.

## Discussion

Although accumulating evidence has shown that KRAS mutations can promote CRC progression, there is still a lack of effective therapy for these subpopulations of patients. Many genes can promote CRC recurrence and prognosis [28]. Therefore, a better investigation of the molecular mechanism underlying KRAS mutant CRC metastasis is still urgently needed [19]. In a previous study, High *miR-196a* levels promote the oncogenic phenotype of CRC, and the *miR-196a* concentrations (240 nmol/L) decreased mRNA levels of HOXA7. However, the function of HOXA7 in CRC was not further investigated in this study [29]. We discovered that HOXA7 was overexpressed in CRC and that high levels of HOXA7 were correlated with a greater incidence of metastasis, more aggressive tumor phenotype, and shorter overall survival time. In addition, HOXA7 overexpression was an independent poor prognostic factor in patients with KRAS mutant CRC but not in those with KRAS wild-type CRC. Furthermore, HOXA7 promoted KRAS mutant CRC metastasis in immunocompetent mice. These results demonstrated the importance of HOXA7 in promoting KRAS mutant CRC metastasis.

Immune invasion is one of the main features of tumors [30] and is frequently associated with PD-1/PD-L1 amplification and the infiltration of immunosuppressive cells, such as MDSCs [31, 32]. MDSCs are representative immunosuppressive cells in various malignant diseases, especially cancers [33]. MDSCs have emerged as the primary impediment to immunotherapy in human CRC, according to increasing data in recent years [34]. More importantly, MDSCs can inhibit CD8^+^ T cell cytotoxicity. In this study, we demonstrated that HOXA7 overexpression leads to immune suppression in KRAS mutant CRC via the chemotaxis of MDSCs. An in vivo study showed that pharmacological depletion of MDSCs with anti-Gr1 antibody treatment can inhibit HOXA7-mediated KRAS mutant CRC metastasis. These findings support the view that depletion of MDSCs can suppress KRAS mutant CRC metastasis. Two major subsets of MDSC have been identified: monocytic (M-MDSC) and polymorphonuclear (PMN-MDSC), which share phenotypic and morphologic features with monocyte and neutrophil, respectively. In some circumstances, M-MDSCs have been reported to possess higher immunosuppressive activity than PMN-MDSCs within the tumor microenvironment. Regretfully, we didn’t further define the MDSCs into M-MDSC and PMN-MDSC [25]. This issue will be further studied in the following study.

AMG510, a KRASG12C inhibitor, showed a long-lasting antitumor response in immunocompetent mice. Nevertheless, its antitumor effect was weak in immunodeficient mice, indicating that T cells are required for AMG510 to function. The combination of AMG510 with anti-PD-1 can enhance CD8^+^ T cell infiltration and produce a durable antitumor response [9]. These studies have laid the foundation for the combination of KRAS inhibitors and immunotherapy, but KRASG12C is a rare type of CRC, accounting for only approximately 3% of all KRAS mutations. Most patients with KRAS mutations in CRC cannot benefit from this combination therapy. Therefore, it has become urgent to investigate combination strategies to improve therapeutic effectiveness for KRAS mutant CRC.

In particular, anti-PD-1/anti-PD-L1 antibody treatment combined with anti-CTLA4 drugs, locoregional therapies, or VEGF/VEGFR inhibitors synergistically improves antitumor immunity [34–36]. These studies suggested that antitumor agents that stimulate tumor immunity in combination with anti-PD-1/anti-PD-L1 antibody treatment might show promising results in tumor treatment. Our in vivo data indicated that combined treatment of SB265610 with anti-PD-L1 can significantly inhibit HOXA7-mediated KRAS mutant CRC metastasis compared with the control or a single agent alone. In addition, the combination therapy significantly reduced MDSC recruitment while increasing CD8^+^ T cell infiltration. These results provide a novel combination therapy for inhibiting HOXA7-induced KRAS mutant CRC metastasis.

In summary, we demonstrated that HOXA7 overexpression upregulated CXCL1 expression, facilitating the recruitment and infiltration of MDSCs into the KRAS mutant CRC tumor niche. Targeting CXCL1 in combination with anti-PD-L1 antibody treatment largely suppressed HOXA7-mediated KRAS mutant CRC metastasis. Our work indicated that HOXA7 is a potential prognostic biomarker for KRAS mutant CRC, and targeting the oncogenic loop may provide a promising therapeutic strategy for HOXA7-mediated KRAS mutant CRC metastasis.

## Supplementary Information

**Additional file 1. **Additional Material and Methods, Figures and tables.

## Data Availability

The datasets used during the current study are available from the corresponding author on reasonable request.

## Abbreviations

CRC: Colorectal cancer
HOXA7: Homeobox A7
HE: Hematoxylin and eosin
IHC: Immunohistochemistry
BLI: Bioluminescent imaging
CXCL1: Chemokine (C-X-C motif) ligand 1
CXCR2: Chemokine (C-X-C motif) receptor 2

## References

[1] Siegel RL, Miller KD, Fedewa SA (2020). Colorectal cancer statistics, 2020. Cancer J Clin.

[2] Parker BS, Rautela J, Hertzog PJ (2016). Antitumour actions of interferons: implications for cancer therapy. Nat Rev Cancer.

[3] Pereira A, Rego J, Morris V (2015). Association between KRAS mutation and lung metastasis in advanced colorectal cancer. Br J Cancer.

[4] Benvenuti S, Sartore BA, Nicolantonio FD (2007). Oncogenic activation of the RAS/RAF signaling pathway impairs the response of metastatic colorectal cancers to anti-epidermal growth factor receptor antibody therapies. Cancer Res.

[5] Bennouna J, Sastre J, Arnold D (2013). Continuation of bevacizumab after first progression in metastatic colorectal cancer (ML18147): a randomised phase 3 trial. Lancet Oncol.

[6] Neeraj L, Brian SW, Ghaleb G (2018). KRAS mutation and consensus molecular subtypes 2 and 3 are independently associated with reduced immune infiltration and reactivity in colorectal cancer. Clin Cancer Res.

[7] Lawrence JL (2021). Perspective on model-informed drug development. CPT Pharmacomet Syst Pharmocol.

[8] Marwam GF, Scott K, Yasutoshi K (2022). Sotorasib for previously treated colorectal cancers with KRAS ^G12C^ mutation (CodeBreaK100): a prespecified analysis of a single-arm, phase 2 trial. Lancet Oncol.

[9] Canon J, Rex K, Saiki AY (2019). The clinical KRAS (G12C) inhibitor AMG 510 drives anti-tumour immunity. Nature.

[10] Ruddle FH, Bartels JL, Bentley KL (1994). Evolution of Hox genes. Annu Rev Genet.

[11] Shah N, Sukumar S (2010). The Hox genes and their roles in oncogenesis. Nat Rev Cancer.

[12] Tang B, Qi G, Sun X (2016). HOXA7 plays a critical role in metastasis of liver cancer associated with activation of Snail. Mol Cancer.

[13] Li JY, Ye M, Zhou CC (2020). Expression profile and prognostic values of HOXA family members in laryngeal squamous cell cancer. Front Oncol.

[14] Wang S, Diao YJ, Zhu BB (2020). MiR-193a-5p suppresses cell proliferation and induces cell apoptosis by regulating HOXA7 in human ovarian cancer. Neoplasma.

[15] Li YM, Liu ZY, Wang JC (2019). RIP3 deficiency recruits myeloid-derived suppressor cells to hepatocellular carcinoma through the CXCL1-CXCR2 axis. Hepatology.

[16] Gabrilovich DI, Ostrand RS, Bronte V (2012). Coordinated regulation of myeloid cells by tumours. Nat Rev Immunol.

[17] Overman MJ, Mcdermott R, Leach JL (2017). Nivolumab in patients with metastatic DNA mismatch repair-deficient or microsatellite instability-high colorectal cancer (CheckMate 142): an open-label, multicentre, phase 2 study. Lancet Oncol.

[18] Le DT, Uram JN, Wang H (2015). PD-1 blockade in tumors with mismatch-repair deficiency. N Engl J Med.

[19] Liao W, Overman MJ, Boutin AT (2019). KRAS-IRF2 axis drives immune suppression and immune therapy resistance in colorectal cancer. Cancer Cell.

[20] Ganesh K, Stadler ZK, Cercek A (2019). Immunotherapy in colorectal cancer: rationale, challenges and potential. Nat Rev Gastroenterol Hepatol.

[21] Dang YZ, Chen J, Feng WB (2020). Interleukin 1β-mediated HOXC10 overexpression promotes hepatocellular carcinoma metastasis by upregulating PDPK1 and VASP. Theranostics.

[22] Wang D, Sun H, Wei J (2017). CXCL1 is critical for premetastatic niche formation and metastasis in colorectal cancer. Cancer Res.

[23] Taki M, Abiko K, Baba T (2018). Snail promotes ovarian cancer progression by recruiting myeloid-derived suppressor cells via CXCR2 ligand upregulation. Nat Common.

[24] Pekarek AL (1995). Inhibition of tumor growth by elimination of granulocytes. J Exp Med.

[25] Liu M, Zhou JY, Liu XY (2020). Targeting monocyte-intrinsic enhancer reprogramming improves immunotherapy efficacy in hepatocellular carcinoma. Gut.

[26] Zhou JY, Liu M, Sun HY (2018). Hepatoma-intrinsic CCRK inhibition diminishes myeloid-derived suppressor cell immunosuppression and enhances immune-checkpoint blockade efficacy. Gut.

[27] He Q, Huang WJ, Liu DF (2021). Homeobox B5 promotes metastasis and poor prognosis in hepatocellular carcinoma, via FGFR4 and CXCL1 upregulation. Theranostics.

[28] Xu GR, Zhang MH, Zhu HX (2017). A 15-gene signature for prediction of colon cancer recurrence and prognosis based on SVM. Gene.

[29] Schimanski CC, Frerichs K, Fareed Rahman F (2009). High *miR-196a* levels promote the oncogenic phenotype of colorectal cancer cells. World J Gastroenterol.

[30] Hanahan D, Weinberg RA (2011). Hallmarks of cancer: the next generation. Cell.

[31] Rebekka W, Viktor F, Hu X (2018). Myeloid-derived suppressor cells hinder the anti-cancer activity of immune checkpoint inhibitors. Front Immunol.

[32] Kaoru A, Masaki M, Junzo H (2013). PD-L1 on tumor cells is induced in ascites and promotes peritoneal dissemination of ovarian cancer through CTL dysfunction. Clin Cancer Res.

[33] Veglia F, Perego M, Gabrilovich D (2018). Myeloid-derived suppressor cells coming of age. Nat Immunol.

[34] Wolchok JD, Chiarion SV, Gonzalez R (2017). Overall survival with combined nivolumab and ipilimumab in advanced melanoma. N Engl J Med.

[35] Victor TS, Rech AJ, Maity A (2015). Radiation and dual checkpoint blockade activates non-redundant immune mechanisms in cancer. Nature.

[36] Xu JM, Zhang Y, Jia R (2019). Anti-PD-1 antibody SHR 1210 combined with apatinib for advanced hepatocellular carcinoma, gastric or esophagogastric junction cancer: an open-label, dose escalation and expansion study. Clin Cancer Res.

