# Supplementary material for: HOXA7 promotes the metastasis of KRAS mutant colorectal cancer by regulating myeloid-derived suppressor cells
Source: Cancer Cell Int. 2022 Feb 19;22:88. doi: 10.1186/s12935-022-02519-9 (PMC8858502; doi:10.1186/s12935-022-02519-9)
Supplement: Supplementary file 1 — Additional file 1. Additional Material and Methods, Figures and tables. [file 12935_2022_2519_MOESM1_ESM.docx]

**HOXA7 Promotes the Metastasis of KRAS Mutant Colorectal Cancer by Regulating Myeloid-derived Suppressor Cells**

Yunzhi Dang^✉^, Jiao Yu, Shuhong Zhao, Ximing Cao, Qing Wang

Department of Radiation Oncology, Shaanxi Provincial People’s Hospital, Xi’an, 710086, China

**Corresponding author**: Yunzhi Dang, Department of Radiation Oncology, Shaanxi Provincial People’s Hospital, Xi’an 710086, China.

**E-mail**: dangyunzhi@xiyi.edu.cn

**Additional Material and Methods**

**Construction of lentivirus and stable cell lines**

Construction of lentivirus and stable cell lines Lentiviral vectors encoding shRNAs were generated using PLKO.1-TRC (Addgene) and designated as LV-shHOX 7 (mice), LV-shHOXA7 (human), and LV-shcontrol. “LV-shcontrol” is a non-target shRNA control. The vector “pLKO.1-puro non-Target shRNA Control Plasmid DNA” (purchased from Sigma, SHC016) contains an shRNA insert that does not target any known genes from any species. Three short hairpin RNAs (shRNAs) sequences were: shHOXA7 (mice), 5’-GAAGTGGAAGAAAGAGCATAA-3’; shHOXA7 (human), 5’-CTTTAAGAGACTCACTGGTTT-3’; shCXCL1: 5’-CCTGCCCTTATAGGAACAGAA-3’

Lentiviral vectors encoding the mice HOXA7 genes were constructed in FUW-teto (Addgene) and designated as LV-HOXA7. An empty vector was used as the negative control and was designated as LV-control. Concentrated lentivirus was transfected into the HCC cells with a multiplicity of infection (MOI) ranging from 30 to 50 in the presence of polybrene (6 μg/ml). Seventy-two hours after infection, HCC cells were selected for 2 weeks using 2.5 μg/ml puromycin (OriGene). Selected pools of knockdown and overexpression cells were used for the follow experiments.

***In vitro* invasion and migration assay**

For the migration and invasion assay, a 24 well chamber with 8 μm pore filter (Corning corporation, USA) was used. For migration assay, 5×10^5^ cells were seeded into the upper chamber in serum-free medium. For invasion assay, 5×10^5^ cells were implanted in the top chamber with Matrigel (Corning corporation, USA). After 24-48 hours, the cells were fixed with 95% ethanol and stained with crystal violet. The mean of triplicate assays for each experimental condition was used.

**Real-time PCR**

Total RNA was extracted using TRIzol Reagent (Invitrogen), and reverse transcription was performed using the Advantage for RT-PCR Kit (Takara) according to the manufacturer’s instructions. For the real-time PCR analysis, aliquots of double-stranded cDNA were amplified using a SYBR Green PCR Kit (Applied Biosystems). For the clinical tissue samples, the fold change of the target gene was determined by the following equation: 2^–ΔΔCt^ (ΔΔCt = ΔCt^tumor^ – ΔCt^nontumor^). This value was normalized to the average fold change in the normal colon tissues, which was defined as 1.0. All reactions were performed in duplicate. The primer sequences for HOXA7 sense were 5’-CCCTGGATGCGGTCTTCA-3’, HOXA7 antisense was 5’-CCTTCGTCCTTATGCTCTTTCT-3’.

**KRAS mutation test**

Genomic DNA of [paraffin-embedded](javascript:;) [tissue](javascript:;) was extracted according to kit (E.Z.N.A, FFPE DNA Kit, Lot.D3399-01, OMEGA, USA) instructions and stored at 20℃ for later use. Mutation analysis of KRAS in cancer within codon 12 and codon 13 (c.38G>A, p.G13D) of exon 2 of KRAS gene was detected as follow:

For the real-time PCR analysis, aliquots of double-stranded cDNA were amplified using a SYBR Green PCR Kit (Applied Biosystems). The cycling parameters were as follow: 95°C for 15 s, 55-60°C for 15 s, and 72°C for 15 s for 45 cycles. A melting curve analysis was then performed. The Ct was measured during the exponential amplification phase, and the amplification plots were analyzed using SDS 1.9.1 software (Applied Biosystems). Then, the sequence analysis was performed using ABI 3730XL sequencer. Analysis the KRAS mutation status by Chromas under SNR＞98% condition.

**Western Blotting**

For Western blotting assay, the lysed cells protein was separated on SDS-PAGE and transferred onto polyvinylidene difluoride membrane. The nonspecific binding was blocked with 10% non-fat milk for one hour. The membranes were incubated with specific antibody overnight at 4°C. Western blotting of β-actin on the same membrane was used as a loading control. Antibody against for HOXA7 (ab211521) expression was purchased from abcam. Antibody against β-actin (A1978) was purchased from sigma. The membranes were then washed with PBS 3 times and incubated with an HRP-conjugated secondary antibody. Proteins were visualized using a Immobilon^TM^ Western Chemiluminescent HRP substrate (Millipore, USA).

**Additional Figure**


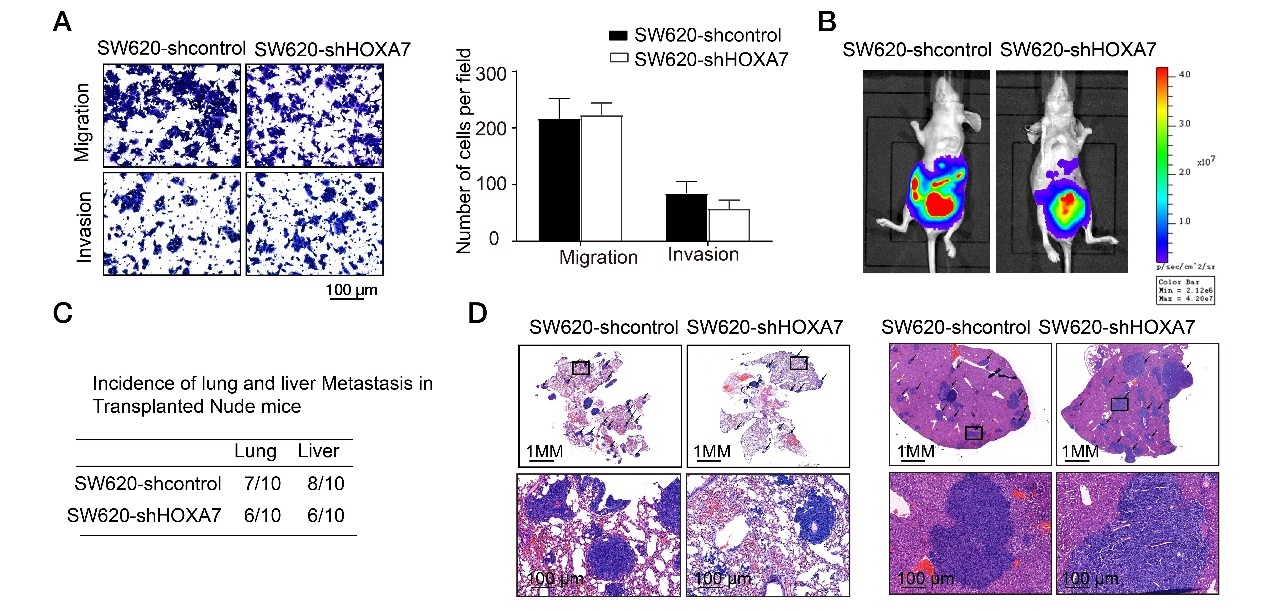


**Figure S1. HOXA7 cannot promote KRAS-mutant CRC metastasis in nude mice.**

(A). Transwell assay analysis of the invasion and metastasis abilities of SW620-shcontrol cells and SW620-shHOXA7 cells.

(B-D). In vivo metastasis assays in nude mice. (B). Bioluminescent images. (C). The incidence of lung metastasis. (D). Representative HE staining.


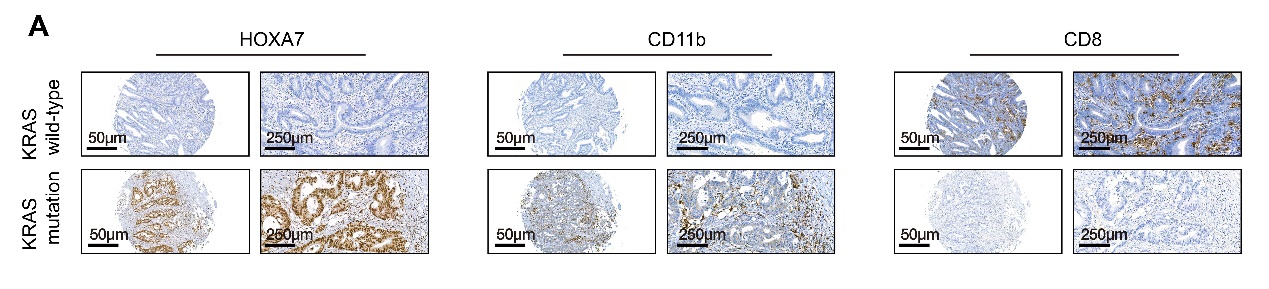


**Figure S2** (A). The expression of HOXA7 and CD11b and CD8 in each group was analyzed by IHC.


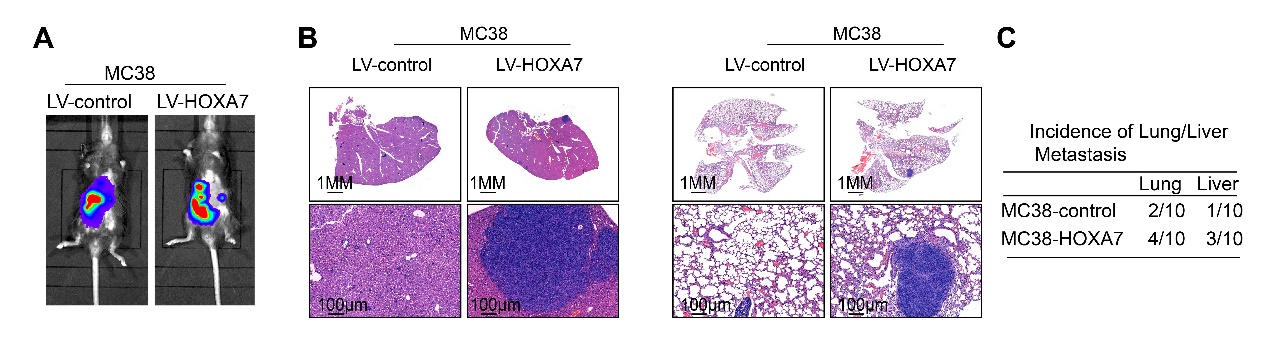


**Figure S3. HOXA7 promotes CRC metastasis in C57BL/6 mice**

(A-C). Metastasis assays of MC38-control and MC38-HOXA7 metastasis ability in the C57BL/6 mice. (A). Bioluminescent imaging. (B). HE. (C). and the incidence of lung and liver metastasis.


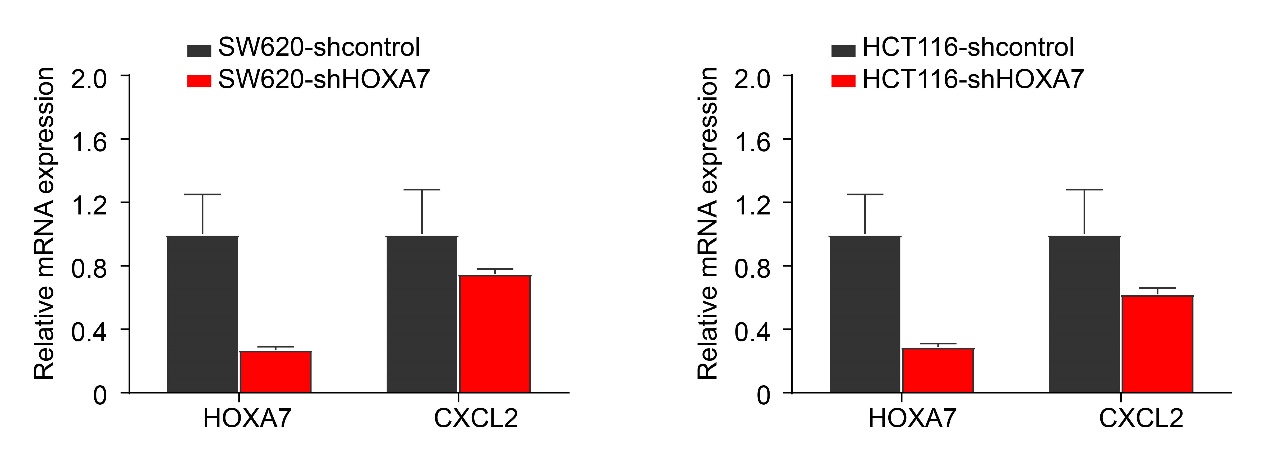


**Figure S4.** HOXA7 and CXCL2 expression in the indicated cells as determined by real-time PCR analysis.

**Additional Table**

**Table S1. Univariate and multivariate analysis of factors associated with survival in cohort of human KRAS-mutant CRC**

| Clinical Variables | **Univariate COX regression analysis** | | **Multivariate COX regression analysis** | |
| --- | --- | --- | --- | --- |
|  | HR (95% CI) | P value | HR (95% CI) | P value |
| Age (≤50 versus > 5)0 | 0.842 (0.490-1.446) | 0.532 |  |  |
| Gender (female vs male) | 1.071 (0.750-1.528) | 0.707 |  |  |
| Tumor size (≤5 cm vs >5 cm) | 0.797 (0.554-1.146) | 0.221 |  |  |
| Tumor differentiation (well/moderate vs poor) | 0.286 (0.196-0.418) | <0.001 | 0.868 (0.542-1.391) | 0.557 |
| Tumor invasion (I-II versus III) | 0.498 (0.333-0.745) | 0.001 | 0.704 (0.454-1.092) | 0.117 |
| Lymph node metastasis (absent vs present) | 0.217 (0.143-0.330) | <0.001 | 2.371 (0.861-6.536) | 0.095 |
| Distant metastasis (absent vs present) | 0.211 (0.135-0.328) | <0.001 | 0.565 (0.338-0.944) | 0.029 |
| TNM stage (I/II vs III/IV) | 0.148 (0.095-0.232) | <0.001 | 0.111 (0.038-0.328) | 0.013 |
| HOXA7 expression (negative vs positive) | 0.203 (0.131-0.313) | <0.001 | 0.374 (0.224-0.625) | <0.001 |

**Table S2. Univariate and multivariate analysis of factors associated with survival in cohort of human KRAS-wild type CRC**

| Clinical Variables | **Univariate COX regression analysis** | | **Multivariate COX regression analysis** | |
| --- | --- | --- | --- | --- |
|  | HR (95% CI) | P value | HR (95% CI) | P value |
| Age (≤50 versus > 50) | 1.336 (0.839-2.126) | 0.222 |  |  |
| Gender (female vs male) | 0.824 (0.572-1.189) | 0.301 |  |  |
| Tumor size (≤5 cm vs >5 cm) | 1.251 (0.862-1.817) | 0.239 |  |  |
| Tumor differentiation (well/moderate vs poor) | 0.134 (0.091-0.197) | <0.001 | 0.637 (0.388-1.046) | 0.075 |
| Tumor invasion (I-II versus III) | 0.244 (0.166-0.359) | <0.001 | 0.258 (0.117-0.570) | 0.001 |
| Lymph node metastasis (absent vs present) | 0.083 (0.054-0.129) | <0.001 | 0.345 (0.155-0.767) | 0.009 |
| Distant metastasis (absent vs present) | 0.111 (0.072-0.172) | <0.001 | 0.508 (0.351-0.737) | <0.001 |
| TNM stage (I/II vs III/IV) | 0.07 3 (0.047-0.113) | <0.001 | 0.490 (0.280-0.858) | 0.013 |
| HOXA7 expression (negative vs positive) | 0.812 (0.557-1.186) | 0.282 | 1.116 (0.760-1.640) | 0.575 |

**Table S3. Chemokines and Receptors RT^2^ Profiler PCR Array of SW620-shHOXA7 vs SW620-shcontrol**

| **Gene** | **Description** | **Fold change** |
| --- | --- | --- |
| CXCL1 | chemokine (C-X-C motif) ligand 1 | -5.40 |
| CXCL1 | chemokine (C-X-C motif) ligand 1 | -3.97 |
| LYZ | lysozyme | -3.49 |
| OPN | osteopontin | -3.45 |
| CXCR2 | [chemokine (C-C motif) receptor 2](https://www.baidu.com/link?url=-kB9oLVKm3u-OjQzJ3n6uf2UAttfublaVvgK-ajwWW_BUjz7ezftbJQGqB2Pmq9hgCYb8S2yRyiopVAYK59b3cO0-5IgEGBjw3bQJtbRX2K&wd=&eqid=9a00e2ac00006e9e00000004616e7a33) | -3.44 |
| VAV3 | vav 3 guanine nucleotide exchange factor | -3.44 |
| C4BPA | complement component 4 binding protein, alpha | -3.40 |
| ADH6 | alcohol dehydrogenase 6 (class V) | -3.05 |
| CXCL3 | chemokine (C-X-C motif) ligand 3 | -3.01 |
| CCL20 | chemokine (C-C motif) ligand 20 | -3.00 |
| EGFR | complement component 4 binding protein, alpha | -3.00 |
| AKR1B10 /// AKR1B15 | aldo-keto reductase family 1, member B10 (aldose reductase) /// aldo-keto reductase family 1, member B15 | -3.00 |
| VNN3 | vanin 3 | -2.98 |
| SLC6A14 | solute carrier family 6 (amino acid transporter), member 14 | -2.86 |
| SULT2A1 | sulfotransferase family, cytosolic, 2A, dehydroepiandrosterone (DHEA)-preferring, member 1 | -2.81 |
| DLG1 | discs, large homolog 1 (Drosophila) | -2.71 |
| ADH6 | alcohol dehydrogenase 6 (class V) | -2.70 |
| AKR1B10 | aldo-keto reductase family 1, member B10 (aldose reductase) | -2.67 |
| CXCL3 | chemokine (C-X-C motif) ligand 3 | -2.67 |
| AKR1B10 | aldo-keto reductase family 1, member B10 (aldose reductase) | -2.66 |
| ANGPTL3 | angiopoietin-like 3 | -2.65 |
| HAL | histidine ammonia-lyase | -2.62 |
| HAL | histidine ammonia-lyase | -2.58 |
| CGA | glycoprotein hormones, alpha polypeptide | -2.56 |
| MCTP1 | multiple C2 domains, transmembrane 1 | -2.53 |
| MAN1A1 | mannosidase, alpha, class 1A, member 1 | -2.53 |
| DEFB1 | defensin, beta 1 | -2.52 |
| ITIH2 | inter-alpha-trypsin inhibitor heavy chain 2 | -2.50 |
| CXCL10 | chemokine (C-X-C motif) ligand 10 | -2.50 |
| KNG1 | kininogen 1 | -2.50 |
| FBXL17 | F-box and leucine-rich repeat protein 17 | -2.49 |
| PAH | phenylalanine hydroxylase | -2.46 |
| SLC6A14 | solute carrier family 6 (amino acid transporter), member 14 | -2.43 |
| CPB2 | carboxypeptidase B2 (plasma) | -2.41 |
| GPX2 | glutathione peroxidase 2 (gastrointestinal) | -2.41 |
| HNRNPU-AS1 | HNRNPU antisense RNA 1 (non-protein coding) | -2.40 |
| ADH4 | alcohol dehydrogenase 4 (class II), pi polypeptide | -2.40 |
| VSNL1 | visinin-like 1 | -2.39 |
| C4BPB | complement component 4 binding protein, beta | -2.39 |
| VSNL1 | visinin-like 1 | -2.37 |
| VNN2 | vanin 2 | -2.36 |
| DLK1 | delta-like 1 homolog (Drosophila) | -2.33 |
| NR1H4 | nuclear receptor subfamily 1, group H, member 4 | -2.31 |
| MCC | mutated in colorectal cancers | -2.30 |
| HLF | hepatic leukemia factor | -2.30 |
| FGB | fibrinogen beta chain | -2.29 |
| CHODL | chondrolectin | -2.29 |
| DEFB1 | defensin, beta 1 | -2.29 |
| CHODL | chondrolectin | -2.28 |
| VNN3 | vanin 3 | -2.27 |
| DLK1 | delta-like 1 homolog (Drosophila) | -2.25 |
| DLK1 | delta-like 1 homolog (Drosophila) | -2.25 |
| MCC | mutated in colorectal cancers | -2.25 |
| C19orf80 | chromosome 19 open reading frame 80 | -2.25 |
| HAL | histidine ammonia-lyase | -2.24 |
| LHFPL2 | lipoma HMGIC fusion partner-like 2 | -2.23 |
| KNG1 | kininogen 1 | -2.23 |
| CHODL | chondrolectin | -2.21 |
| GK | glycerol kinase | -2.21 |
| FAM176A | family with sequence similarity 176, member A | -2.20 |
| CXCL2 | chemokine (C-X-C motif) ligand 2 | -2.19 |
| SLC4A4 | solute carrier family 4, sodium bicarbonate cotransporter, member 4 | -2.16 |
| ITIH2 | inter-alpha-trypsin inhibitor heavy chain 2 | -2.13 |
| SAA2-SAA4 /// SAA4 | SAA2-SAA4 readthrough /// serum amyloid A4, constitutive | -2.13 |
| CXCL5 GLYR1 | chemokine (C-X-C motif) ligand 5 /// glyoxylate reductase 1 homolog (Arabidopsis) | -2.12 |
| NT5E | 5'-nucleotidase, ecto (CD73) | -2.12 |
| SNTB1 | syntrophin, beta 1 (dystrophin-associated protein A1, 59kDa, basic component 1) | -2.11 |
| SCG5 | secretogranin V (7B2 protein) | -2.11 |
| HABP2 | hyaluronan binding protein 2 | -2.10 |
| NT5E | 5'-nucleotidase, ecto (CD73) | -2.08 |
| ENPP2 | ectonucleotide pyrophosphatase/phosphodiesterase 2 | -2.08 |
| VNN2 | vanin 2 | -2.07 |
| KRT23 | keratin 23 (histone deacetylase inducible) | -2.05 |
| CAPRIN2 | caprin family member 2 | -2.04 |
| MBNL3 | muscleblind-like splicing regulator 3 | -2.02 |
| APOA1 | apolipoprotein A-I | -2.02 |
| FRAS1 | Fraser syndrome 1 | -2.01 |
| LTB | lymphotoxin beta (TNF superfamily, member 3) | -2.00 |
| FOXC1 | forkhead box C1 | -1.98 |
| MYL9 | myosin, light chain 9, regulatory | 2.01 |
| CA9 | carbonic anhydrase IX | 2.02 |
| GPR133 | G protein-coupled receptor 133 | 2.03 |
| COL1A2 | collagen, type I, alpha 2 | 2.03 |
| TAGLN | transgenic | 2.05 |
| NEXN | nexilin (F actin binding protein) | 2.08 |
| TAGLN | transgelin | 2.09 |
| TUBA1A | tubulin, alpha 1a | 2.11 |
| COL12A1 | collagen, type XII, alpha 1 | 2.11 |
| TUBA1A | tubulin, alpha 1a | 2.12 |
| FBN1 | fibrillin 1 | 2.12 |
| WIF1 | WNT inhibitory factor 1 | 2.33 |
| JUP /// KRT17 | junction plakoglobin /// keratin 17 | 2.36 |
| TMEM158 | transmembrane protein 158 (gene/pseudogene) | 2.45 |
| PAX6 | paired box 6 | 2.48 |
| AKNA | AT-hook transcription factor | 2.53 |
| COL1A2 | collagen, type I, alpha 2 | 2.53 |
| BMP6 | bone morphogenetic protein 6 | 2.60 |
| ACTA1 | actin, alpha 1, skeletal muscle | 3.39 |
| ACTA1 | actin, alpha 1, skeletal muscle | 3.67 |
